# Time-of-Day Effects on Metabolic and Clock-Related Adjustments to Cold

**DOI:** 10.3389/fendo.2018.00199

**Published:** 2018-04-26

**Authors:** Frederico Sander Mansur Machado, Zhi Zhang, Yan Su, Paul de Goede, Remi Jansen, Ewout Foppen, Cândido Celso Coimbra, Andries Kalsbeek

**Affiliations:** ^1^Department of Endocrinology and Metabolism, Academic Medical Center, University of Amsterdam, Amsterdam, Netherlands; ^2^Hypothalamic Integration Mechanisms, Netherlands Institute for Neuroscience, Amsterdam, Netherlands; ^3^Department of Physiology and Biophysics, Biological Sciences Institute, Universidade Federal de Minas Gerais, Belo Horizonte, Brazil; ^4^Laboratory of Endocrinology, Department of Clinical Chemistry, Academic Medical Centre, University of Amsterdam, Amsterdam, Netherlands

**Keywords:** circadian, locomotor activity, thermoregulation, thermogenesis, gene expression, skeletal muscle, brown adipose tissue

## Abstract

**Background:**

Daily cyclic changes in environmental conditions are key signals for anticipatory and adaptive adjustments of most living species, including mammals. Lower ambient temperature stimulates the thermogenic activity of brown adipose tissue (BAT) and skeletal muscle. Given that the molecular components of the endogenous biological clock interact with thermal and metabolic mechanisms directly involved in the defense of body temperature, the present study evaluated the differential homeostatic responses to a cold stimulus at distinct time-windows of the light/dark-cycle.

**Methods:**

Male Wistar rats were subjected to a single episode of 3 h cold ambient temperature (4°C) at one of 6 time-points starting at Zeitgeber Times 3, 7, 11, 15, 19, and 23. Metabolic rate, core body temperature, locomotor activity (LA), feeding, and drinking behaviors were recorded during control and cold conditions at each time-point. Immediately after the stimulus, rats were euthanized and both the soleus and BAT were collected for real-time PCR.

**Results:**

During the light phase (i.e., inactive phase), cold exposure resulted in a slight hyperthermia (*p* < 0.001). Light phase cold exposure also increased metabolic rate and LA (*p* < 0.001). In addition, the prevalence of fat oxidative metabolism was attenuated during the inactive phase (*p* < 0.001). These metabolic changes were accompanied by time-of-day and tissue-specific changes in core clock gene expression, such as DBP (*p* < 0.0001) and REV-ERBα (*p* < 0.01) in the BAT and CLOCK (*p* < 0.05), PER2 (*p* < 0.05), CRY1 (*p* < 0.05), CRY2 (*p* < 0.01), and REV-ERBα (*p* < 0.05) in the soleus skeletal muscle. Moreover, genes involved in substrate oxidation and thermogenesis were affected in a time-of-day and tissue-specific manner by cold exposure.

**Conclusion:**

The time-of-day modulation of substrate mobilization and oxidation during cold exposure provides a clear example of the circadian modulation of physiological and metabolic responses. Interestingly, after cold exposure, time-of-day mostly affected circadian clock gene expression in the soleus muscle, despite comparable changes in LA over the light–dark-cycle. The current findings add further evidence for tissue-specific actions of the internal clock in different peripheral organs such as skeletal muscle and BAT.

## Introduction

Daily cyclic changes in environmental conditions are key signals for the adaptive and anticipatory activity of most living species, including mammals. The mammalian thermoregulatory system is fairly adapted to periodic changes in ambient temperatures that may reach high amplitudes depending on the geographic location (1–3). Intriguingly, in mammals, a role for daily body temperature cycles in the internal synchronization has been demonstrated, *in vivo, ex vivo*, as well as *in vitro* (1, 4, 5), indicating that the circadian timing system and the thermoregulatory system reciprocally influence each other.

In general, during the dark phase, ambient temperature decreases to its lowest daily levels. Lower ambient temperatures induce metabolic changes aimed to defend internal body temperature. Considering this, it is well known that a cold environment induces autonomic, cardiovascular, metabolic, and behavioral adjustments that depend on the synchronized activation of multiple independent pathways resulting in thermal adaptation/acclimation (6). These adjustments include physiological changes such as vasoconstriction (heat retention or storage) and thermogenesis from both the activation of brown adipose tissue (BAT) (non-shivering) and skeletal muscle involuntary contraction (shivering) (6). Synchronized activation of the autonomic innervation to BAT and white adipose tissue (WAT), liver, adrenal, and skeletal muscle is necessary to produce the necessary amount of energy and heat to keep body temperature within safe levels during cold exposure (7).

Contractile skeletal muscle activity acts as an important heat source during environmental cold exposure in rodents (6, 8) and humans (9, 10). Increased ADP/ATP ratio, 5′adenosine monophosphate-activated protein kinase (AMPK) activity, peroxisome proliferator-activated receptor gamma coactivator 1-alpha (PGC1-α) activity, and consequently, free-fatty acid (FFA) uptake and oxidation, increases intracellular substrate availability for heat production (11). Notably, in BAT, the requirement of a functional molecular clock has been demonstrated, as a deficiency in the expression of the BMAL-1 and PER2 clock gene leads to activation of compensatory heat production mechanisms (12, 13). In humans, it has recently been demonstrated that BAT glucose uptake might be associated with the heat production rhythm (14). The latter data indicate that the central body clock has an important role in the tuning of the cold-evoked response, probably by setting the basal metabolic rate to a new reference level or balancing the autonomic tonus.

Metabolic rate increases in response to cold through shivering and non-shivering thermogenesis. Both processes are centrally regulated (6) and result in increased lipid and carbohydrate oxidation in involved tissues (mainly skeletal muscle and BAT) (8, 15). Therefore, lipid mobilization (from WAT *lipolysis* and liver *de novo* lipogenesis) increases to provide the main substrate used for the thermogenic activity. Interestingly, the molecular clock influences substrate oxidation (16, 17). Therefore, modulation of the central thermoregulatory pathways by the biological clock in the suprachiasmatic nuclei (SCN) might result in changes in cold exposure adjustments depending on the time-of-day. In line with this hypothesis, the master clock projects to the major brain areas involved in metabolic/thermal balance (18, 19).

Core components of the molecular circadian clock are expressed throughout the body, both centrally and peripherally and interact with intracellular pathways directly related to metabolism and heat production (12–14, 16, 17, 20). In view of the above, the present study aimed to investigate how time-of-day modulates the peripheral adjustments induced by cold exposure at a physiological and a molecular level.

## Materials and Methods

### Animals

All experiments were performed in adult male Wistar rats (Charles River Breeding Laboratories, Sulzfeld, Germany). After arrival at the animal facility, animals were housed in individual cages (25 cm × 25 cm × 35 cm), with a 12/12-h light–dark (L/D) schedule [lights on at 0700 h, defined as Zeitgeber Time 0 (ZT0)]. Animals were allowed to adapt to the new environment for 1.5 weeks before the first experiments. All rats were kept under constant temperature (22 ± 2°C) and humidity (50 ± 5%) conditions. Food and water were available *ad libitum*. The animal care committee of the Royal Netherlands Academy of Arts and Sciences (DEC/KNAW) approved all experiments.

### Experimental Procedures

To verify where would be a good site to monitor internal body temperature, a pilot study group was completed before the described experiments. In this preliminary group, each animal was implanted with two data loggers: one in the dorsal subcutaneous area, caudal to the BAT and the other one inside the peritoneal cavity, stitched to the abdominal wall. Since these loggers are not radio-telemetry based, interference between them was not observed, as it would be expected for other available models. After recovery from surgery, animals were exposed to episodes of lowered ambient temperatures during light and dark phases of the L/D cycle. With this initial study, we were able to identify that the intraperitoneal loggers produced the most stable and reliable results.

Experimental animals were anesthetized with isoflurane, which guaranteed a rapid recovery from the small surgery necessary to insert the temperature sensors. A ventral incision at the *linea alba* was made to introduce a data-logger probe (DST nano-T, StarOddi, Iceland) into the peritoneal cavity. Each logger was sutured to the inner musculature before the incision was closed. This procedure allowed continuous monitoring of core body temperature (T_core_) with a decreased risk of internal displacement of the sensor, which could cause misleading readings due to its position. These probes recorded internal T_core_ with 5-min intervals.

**Figure 1:**
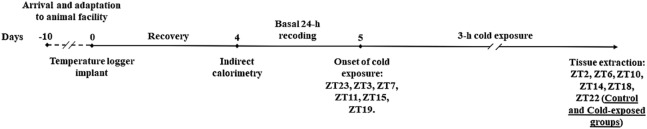
Graphical representation of experimental paradigm.

For the main study (presented in Figure 1), on the fourth day after surgery, the basal 24-h locomotor activity (LA), energy expenditure (EE), and food and water intake were continuously recorded for each animal with an indirect calorimetry system (PhenoMaster/LabMaster, TSE Systems, Bad Homburg, Germany). LA was assessed as beam-breaks recorded during 15 min intervals. Oxygen consumption (VO_2_) and carbon dioxide production (VCO_2_) were assessed every 15 min for 100 s. Respiratory exchange ratio (RER) was calculated according to the formula: VCO_2_/VO_2_. EE was calculated with the formula: (CVO_2_ × VO_2_ + CVCO_2_ × VCO_2_)/1,000, considering CVO_2_ and CVCO_2_ preset reference values given by the manufacturer (CVO_2_ = 3.941 and CVCO_2_ = 1.106). Carbohydrate (CHO) and lipid oxidation were calculated with the adapted *formulae* (21, 22) previously used (23): (4.585 × VCO_2_ − 3.226 × VO_2_)/60,000 for CHO oxidation and (1.695 × VO_2_ − 1.701 × VCO_2_)/60,000 for lipid oxidation. After the basal recordings, the ambient temperature was lowered to 0–5°C at different times of the day: ZT23–2, ZT3–6, ZT7–10, ZT11–14, ZT15–18, and ZT19–22. The average rate of ambient cooling was −0.8°C/10 min during the period of 3 h of cooling activity. Peak rates of −2.3°C/10 min were observed during the first 45 min of the experimental protocol, while rates lower than −0.5°C/10 min were observed and maintained after 90 min of cooling. By the end of each episode of lowered ambient temperature, animals were rapidly anesthetized with 80% CO_2_ and immediately sacrificed by decapitation. For room temperature controls, animals were kept in the same experimental room, but outside the temperature controlled TSE chamber without any previous manipulation and were sacrificed at the same time points. After decapitation, the brain was removed, snap frozen on dry ice, and stored at −80°C. Soleus skeletal muscle and interscapular BAT were collected, frozen in liquid nitrogen, and then stored at −80°C.

### Tissue Processing

#### RNA Extraction and cDNA Synthesis

Total RNA was extracted and purified with the TRIzol reagent protocol (Macherey-Nagel, Oensingen, Switzerland). The quality of RNA was examined by Agilent 2100 Bioanalyzer equipped with Nano chips (Agilent Technologies, Palo Alto, CA, USA) and concentrations were determined by Nanodrop spectrophotometer (ThermoScientific Technologies, Wilmington, DE, USA). A fixed amount of total RNA was reverse-transcribed with SensiFAST cDNA Synthesis Kit (Bioline, Taunton, MA, USA). For the control of genomic DNA contamination, we employed a minus reverse transcriptase sample (−RT).

#### Real-Time PCR (RT-PCR)

The expression of clock, metabolic, and thermogenesis-related genes was evaluated by RT-PCR (LightCycler ^®^ 480, Roche) with the following reaction system: 2 µl of cDNA was incubated with 50 ng of both reverse and forward primer from gene of interest (see Table 1 for primer sequences) and SensiFAST no-ROX Mix (Bioline, Taunton, MA, USA) for a final volume of 10 µl. The relative amount of each gene was normalized against the geometric mean of three housekeeping genes: hypoxanthine-guanine phosphoribosyl transferase (HPRT), ribosomal protein S18 (S18), and glyceraldehyde-3-phosphate dehydrogenase (GAPDH) for the BAT; GAPDH, S18, and cyclophilin for the soleus muscle. The reference genes mentioned (HPRT, GAPDH, S18, and cyclophilin) were selected based on their constant expression under the various experimental conditions (i.e., time of the day and/or ambient temperature). The relative expression level of clock genes and other genes of interest in each sample was obtained by dividing the absolute amount of the target gene by the average of the reference genes values. Each gene/tissue RT-PCR was performed in a single plate.

**Table 1:** Information about gene primers.

Genes	Reference no.	Reverse	Forward
**Clock genes**			
PER1	NM_001034125.1	TGGCCAGGATCTTGAACACTGCTA	ATGCAGAAACAACAGCCACGGTTC
PER2	NM_031678.1	CAACGCCAAGGAGCTCAAGT	CACCCTGAAAAGAAAGTGCGA
CRY1	NM_198750.2	TCATCATGGTCGTCGGACAGA	AAGTCATCGTGCGCATTTCA
CRY2	NM_133405.2	TGTACAAGTCCCACAGGCGGTA	TGGATAAGCACTTGGAACGGAA
ARNLT/BMAL-1	NM_024362.2	TGCAGTGTCCGAGGAAGATAGC	CCGATGACGAACTGAAACACCT
CLOCK	NM_021856.2	TTGCAGCTTGAGACATCGCT	CGATCACAGCCCAACTCCTT
DBP	NM_012543.3	TGCCTTCTTCATGATTGGCTG	CCTTTGAACCTGATCCGGCT
REV-ERBα	NM_001113422.1	CATGGGCATAGGTGAAGATTTCT	ACAGCTGACACCACCCAGATC

**Metabolic genes**			
CREB1	NM_031017.1	ACTCTGCTGGTTGTCTGCTC	GCAGTGACTGAGGAGCTTGT
PGC1-α	NM_031347.1	GGTCATTTGGTGACTCTGG	TGCCATTGTTAAGACCGAG
PGC1-β	NM_176075.2	AGGAGGGCTCATTGCGTTTT	AAAAGGCCATCGGTGAAGGT
PPAR-α	NM_013196.1	GGCCTTGACCTTGTTCATGT	TCACACAATGCAATCCGTTT
PPAR-γ	NM_013124.3	GGGGGTGATATGTTTGAACTTG	CAGGAAAGACAACAGACAAATCA
HSP90	NM_001004082.3	ACCGAATCTTGTCCAGGGCATCA	CGGGCCCACCCTGCTCTGTA
UCP1	NM_012682.2	GCTTTGTGCTTGCATTCTGA	AATCAGCTTTGCTTCCCTCA
UCP2	NM_019354.3	GGGCACCTGTGGTGCTAC	GACTCTGTAAAGCAGTTCTACACCAA
UCP3	NM_013167.2	ATAGTCAGGATGGTACCGAGCA	GCACTGCAGCCTGTTTTGCTGA
CIRBP	NM_031147.2	TAACCACCACCCCTCCAGAA	GCGTTAGGAAGCTTGGGTGT
CPT1-α	NM_031559.2	AAAGACTGGCGCTGCTCA	ACAATGGGACATTCCAGGAG
CPT1-β	NM_013200.1	TGCTTGACGGATGTGGTTCC	GTGCTGGAGGTGGCTTTGGT-
AMPK	NM_019142.2	TAGAGAATGACCCCGCTGCT	TGTCACAGGCATATGGTGGTC
NAMPT	NM_177928.3	TCGACACTATCAGGTGTCTCAG	ACAGATACTGTGGCGGGAATTGCT
FAT/CD36	NM_001109218.1	CCTTGGCTAAATAACGAACTCTG	ACAGTTTTGGATCTTTGACGTG
GLUT4	NM_012751.1	CAGCGAGGCAAGGCTAGA	GGGCTGTGAGTGAGTGCTTTC
HSL	NM_012859.1	CCACCCGTAAAGAGGGAACT	TCACGCTACATAAAGGCTGCT
LPL	NM_012598.2	AGCAATTCCCCGATGTCCA	CAAAACAACCAGGCCTTCGA
ADR-β2	NM_012739.3	CGACCGCTATGAGCGTGTAG	CGCTTCACGTTCGTGCTGGC
ADR-β3	NM_013108.2	CCTTGCTAGATCTCCATGG	CTTCCCAGCTAGCCCTGTT
GR	NM_012576	GGAGCAAAGCAGAGCAGGTTT	ACCTGGATGACCAAATGACCC
FOXO-1	NM_001191846.2	GTAGGGACAGATTGTGGCGAA	ACGAGTGGATGGTGAAGAGTG
ACC1	NM_022193.1	CAGGCTACCATGCCAATCTC	GATGATCAAGGCCAGCTTGT
ACC2	NM_053922.1	GCTTCCGCTCCAGGGTAGAGT	GCACGAGATTGCTTTCCTAG
mTOR	NM_019906.1	CCCGAGGAATCATACAGGTG	AGCAGCATGGGGTTTAGGT
CamK2a	NM_012920.1	AAGGCTGTCATTCCAGGGTC	TGGCGTGAAGGAATCCTCTG

**Housekeeping genes**			
S18	NM_213557.1	TGGCCAGAACCTGGCTATACTTCC	CTCTTCCACAGGAGGCCTACACG
HPRT1	NM_012583.2	AACAAAGTCTGGCCTGTATCCAA	GCAGTACAGCCCCAAAATGG
GAPDH	NM_017008.4	TCCACCACCCTGTTGCTGTA	TGAACGGGAAGCTCACTGG
Cyclophilin	NM_017101.1	GAAGGAATGGTTTGATGGGT	ATGTGGTCTTTGGGAAGGTG

### Statistical Analysis

The effects of cold exposure and duration in T_core_, LA, VO_2_, RER, EE, CHO, and lipid oxidation, as well as food and water intake, were assessed with a repeated measures ANOVA two-way followed by an appropriate *post hoc* test. The net area under the curve (AUC) for each condition and parameter was calculated, i.e., the absolute change of the physiological variable during the analyzed experimental period compared to *t* = 0. “Net” AUC in this case means that the area of the negative peaks (i.e., decrease compared to *t* = 0) was subtracted from that of the positive peaks (i.e., increases compared to *t* = 0). To analyze the combined effect of cold exposure and time-of-day an ANOVA two-way was applied to the AUC data. The time-of-day effect was assessed with an independent ANOVA one-way for control and cold-exposed situations followed by *post hoc* analysis when appropriate. The effects of cold exposure and time-of-day on mRNA expression were assessed with ANOVA two-way followed by an appropriate *post hoc* test. Data are expressed as mean ± SEM. Differences were considered statistically significant at *p* < 0.05.

## Results

### Daily Oscillations of Body Temperature, LA, and Energy Metabolism

**Figure 2:**
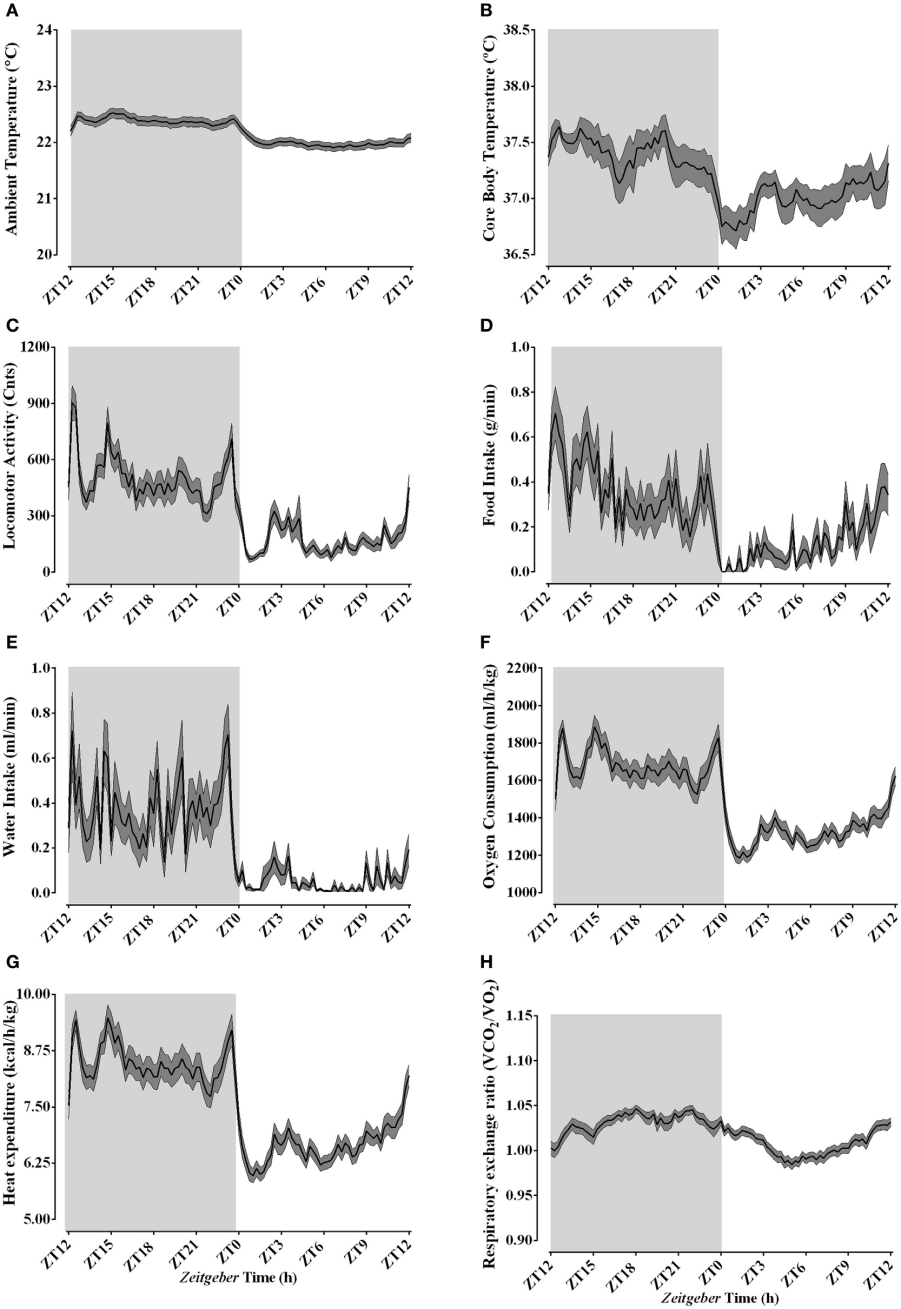
Daily rhythms of thermal, behavioral, and metabolic parameters under regular animal facility conditions. Light phase begins at Zeitgeber Time 0 (ZT0) and dark phase (shaded areas) begins at ZT12. Regular daily oscillatory levels of the ambient temperature **(A)**, body temperature **(B)**, locomotor activity **(C)**, food intake **(D)**, water intake **(E)**, oxygen consumption **(F)**, heat production **(G)**, and respiratory quotient **(H)** were monitored approximately 24 h before the cold episodes. Results are presented as mean ± SEM (*n* = 34–50).

During the basal data collection, 24 h before the actual experiment, ambient temperature inside the calorimetric chamber was kept close to the typical animal facility temperature of 22.18 ± 0.21°C (Figure 2A), although a time-of-day effect was apparent (Table 2). Animals exhibited a clear day/night oscillation in all the studied variables, despite the high variability observed for both food and water intake (Table 2; Figure 2). Core body temperature (Figure 2B) and spontaneous LA (Figure 2C) peaked during the active phase. The increased activity was associated with increased food and water intake (Figures 2D,E) and resulted in augmented oxygen consumption (Figure 2F) and heat production (Figure 2G). During the active phase, substrate utilization was shifted toward carbohydrate oxidation, while during the rest phase, lipid metabolism was predominant as observed through RER (Figure 2H). The baseline levels of thermal, behavioral, and metabolic parameters at the onset of our cooling paradigm were similar to those observed during the previous day and both exhibited time of the day related changes (Table S1 in Supplementary Material).

**Table 2:** Mean basal 24-h and light/dark levels of thermal, behavioral, and metabolic parameters under control conditions (24 h before environmental cooling).

	Mean_24-h_ ± SD	Mean_light phase_ ± SD	Mean_dark phase_ ± SD	*n*	Time-of-day effect	*p* Value
Ambient temperature (°C)	22.18 ± 0.21	21.99 ± 0.07	22.39 ± 0.06	50	*F*(96, 4.753) = 7.059	<0.0001
Core body temperature (°C)	37.20 ± 0.25	37.00 ± 0.14	37.40 ± 0.14	34	*F*(96, 3.201) = 2.695	<0.0001
Locomotor activity (Cnts)	337.1 ± 194.8	175.5 ± 81.1	502.1 ± 125.6	50	*F*(96, 4.753) = 12.54	<0.0001
Oxygen consumption (ml/h/kg)	1,500 ± 196.1	1,327 ± 86.65	1,677 ± 86.64	50	*F*(96, 4.753) = 14.44	<0.0001
Respiratory exchange ratio (VO_2_/CO_2_)	1.02 ± 0.02	1.03 ± 0.01	1.01 ± 0.01	50	*F*(96, 4.753) = 8.036	<0.0001
Heat Expenditure (kcal/h/kg)	7.56 ± 1.00	6.67 ± 0.44	8.47 ± 0.42	50	*F*(96, 4.753) = 15.51	<0.0001
Food intake (g/15 min)	0.24 ± 0.17	0.12 ± 0.10	0.36 ± 0.12	50	*F*(96, 4.749) = 4.241	<0.0001
Water intake (ml/15 min)	0.21 = 0.19	0.05 ± 0.05	0.37 ± 0.15	49	*F*(96, 4.749) = 5.263	<0.0001

### Effect of Time-of-Day on Body Temperature and EE During Acute Environmental Cooling

**Figure 3:**
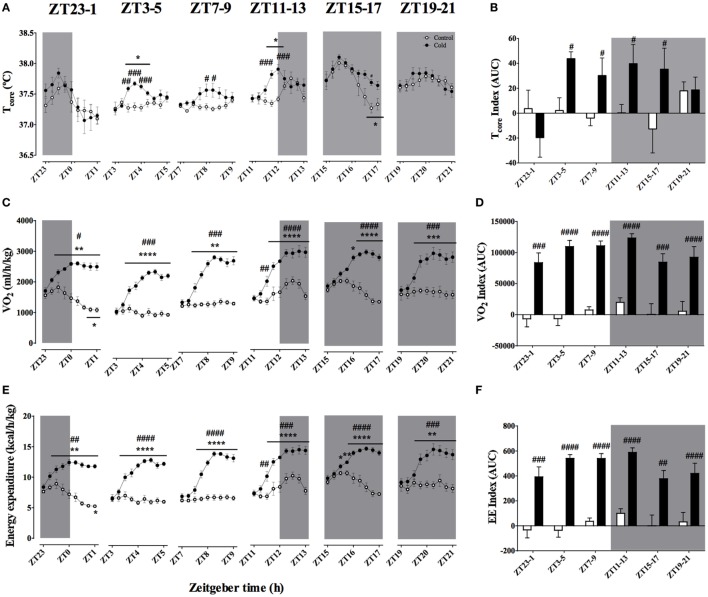
Effect of time-of-day on body temperature **(A,B)**, VO_2_ **(C,D)**, and energy expenditure **(E,F)** changes induced by environmental cooling. Results are presented as averages of 15 min bins during 2 h of environmental cooling **(A,C,E)** and as the area under the curve (AUC) **(B,D,F)** calculated as the net change from the basal levels before cold exposure. Light phase began at Zeitgeber Time 0 (ZT0) and dark phase (shaded areas) began on ZT12. Results are presented as mean ± SEM. * indicates differences between the first and the other time-points within the control or the cold-exposed group, *p* < 0.05. ^#^ indicates differences between control and cold conditions, *p* < 0.05. *n* = 5–11/group/ZT.

Average ambient temperature after 2 h of cooling was 4.01 ± 0.07°C, regardless of time of the day. Body temperature was affected by cold exposure in a time-of-day-dependent fashion (Figures 3A,B). When experimental cooling started during the light phase (ZT3, 7, and 11), a slight and transient hyperthermia was observed (*p* < 0.001). Interestingly, during the ZT15–18 protocol, core body temperature stayed elevated during the final 120 min of cold exposure (*p* < 0.001), despite the normal decrease in this parameter observed in the control animals. Cold exposure during the ZT19–22 and ZT23–2 protocols did not affect core body temperature as compared to baseline values. The time-of-day-dependent body temperature responses were confirmed by the AUC analysis (Figure 3B; Table 3; Table S2 in Supplementary Material), showing a significant effect of both ZT and cold exposure.

**Table 3:** ANOVA two-way table (ZT × ambient temperature) demonstrating the effect of cold exposure and time of the day on net area under the curve (in arbitrary units for each parameter) for thermal, behavioral, and metabolic parameters.

	Effect of ZT		Effect of cold		Interaction	
	*F*(DFn, DFd)	*p* Value	*F*(DFn, DFd)	*p* Value	*F*(DFn, DFd)	*p* Value
Body temperature	*F*(5, 56) = 3.734	0.0055	*F*(1, 56) = 4.487	0.0386	*F*(5, 56) = 2.183	0.0689
Locomotor activity	*F*(5, 74) = 0.372	0.8665	*F*(1, 74) = 42.21	<0.0001	*F*(5, 74) = 0.839	0.5265
VO_2_	*F*(5, 88) = 3.743	0.0040	*F*(1, 88) = 201.2	<0.0001	*F*(5, 88) = 0.3105	0.9055
Respiratory exchange ratio	*F*(5, 88) = 3.404	0.0074	*F*(1, 88) = 107.6	<0.0001	*F*(5, 88) = 5.558	0.0002
CHO oxidation	*F*(5, 88) = 3.314	0.0087	*F*(1, 88) = 1.619	0.2067	*F*(5, 88) = 4.435	0.0012
Lipid oxidation	*F*(5, 88) = 1.051	0.3931	*F*(1, 88) = 72.19	<0.0001	*F*(5, 88) = 2.017	0.0839
Energy expenditure	*F*(5, 88) = 3.889	0.0031	*F*(1, 88) = 230.2	<0.0001	*F*(5, 88) = 2.405	0.0430
Food intake	*F*(5, 86) = 0.074	0.9960	*F*(1, 86) = 2.434	0.1224	*F*(5, 86) = 1.018	0.4123
Water intake	*F*(5, 88) = 1.065	0.3851	*F*(1, 88) = 0.037	0.8470	*F*(5, 88) = 1.433	0.2203

Body temperature is the result of heat production and heat dissipation. Therefore, understanding heat production dynamics during cold exposure at different phases of the light/dark daily cycle would be of value for the present analysis. A good proxy for heat production is oxygen consumption. Oxygen consumption showed a strong increase during cold exposure (*p* < 0.0001; Figures 3C,D), however, despite the observed effect of ZT, there was no interaction between the two factors (Table 3). As soon as the ambient temperature dropped, VO_2_ rose until reaching a steady state, usually about 60 min after the start of the protocol (*p* < 0.0001). Together with the rise in metabolic rate (VO_2_), EE significantly increased with cold exposure (Figures 3E,F), independent of the time-of-day (Figure 3F; Table 3).

### Effect of Time-of-Day on LA and Food/Water Intake During Acute Environmental Cooling

**Figure 4:**
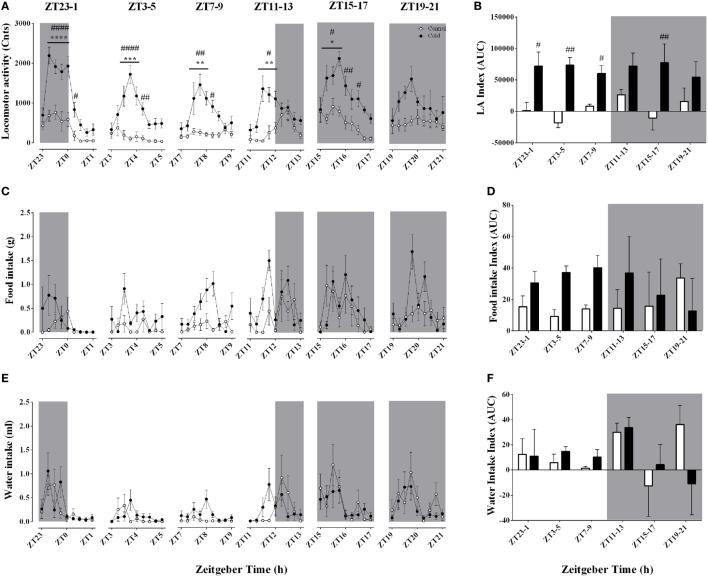
Effect of time-of-day on locomotor activity **(A,B)**, food intake **(C,D)**, and water intake **(E,F)** changes induced by environmental cooling. Results are presented as averages of 15 min bins during 2 h of environmental cooling **(A,C,E)** and area under the curve (AUC) **(B,D,F)** calculated as the net change from the basal levels before cold exposure. Light phase began at ZT and dark phase (shaded areas) began on ZT12. Results are presented as mean ± SEM. * indicates differences between the first and the other time-points within the control or the cold-exposed group, *p* < 0.05. ^#^ indicates differences between control and cold conditions, *p* < 0.05*. n* = 7–11/group/ZT.

In view of the observed effects of time-of-day on the changes in body temperature and heat production in response to a cold environment, it was necessary to investigate whether cold-induced changes in LA or food and/or water intake could play a role. Despite their intrinsic relationship, the changes in T_core_ were not strictly accompanied by changes in LA in a time-of-day-dependent manner (Figures 4A,B; Table 3). Cold exposure induced an increase in LA (Figures 4A,B) that was independent of *Zeitgeber* time (Table 3; Table S2 in Supplementary Material). Regarding food and water intake, regardless of time-of-day cold exposure did not affect these parameters (Figures 4C–F; Table 3).

### Effect of Time-of-Day on Respiratory Quotient and Substrate Oxidation During Acute Environmental Cooling

**Figure 5:**
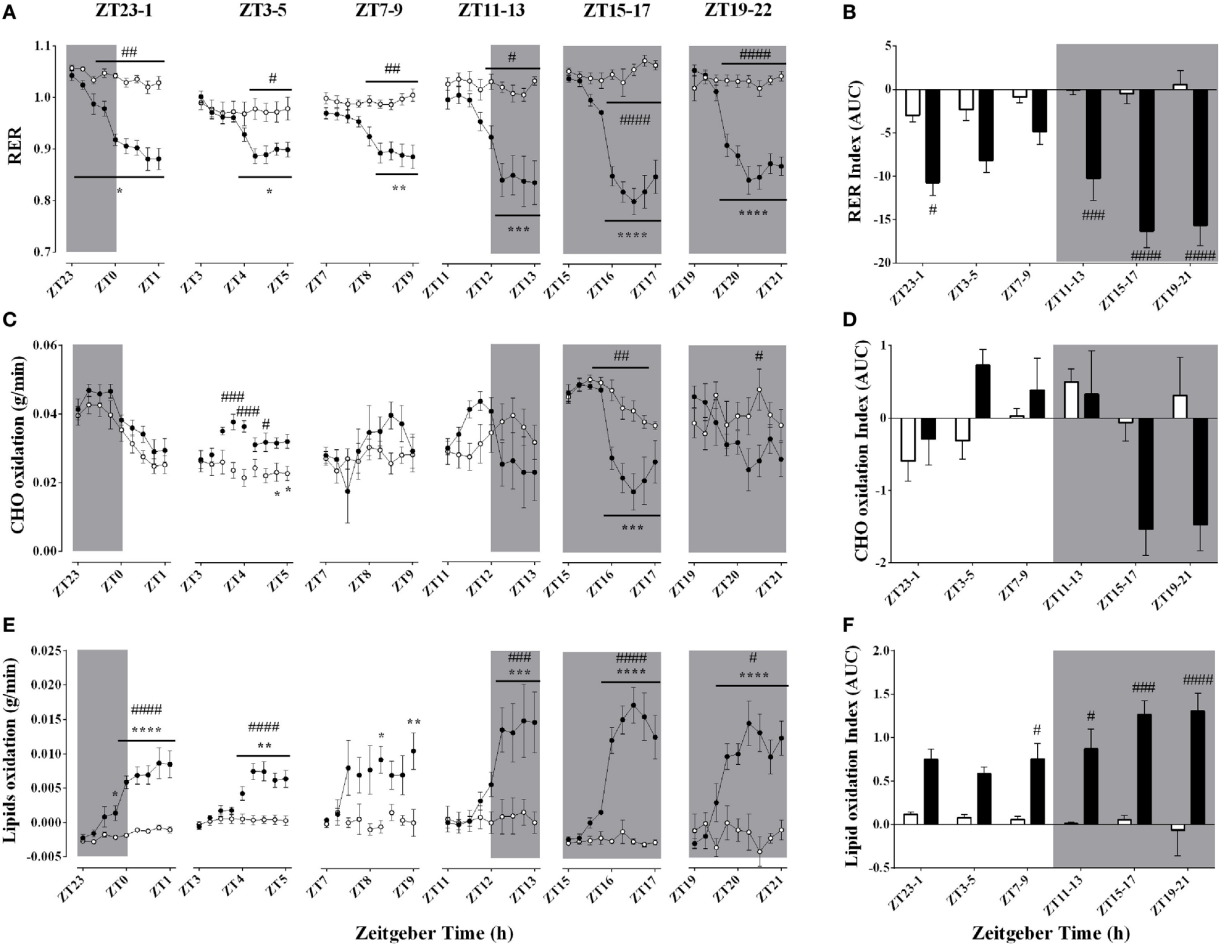
Effect of time-of-day on respiratory quotient **(A,B)**, carbohydrate **(C,D)**, and lipid oxidation **(E,F)** changes induced by environmental cooling. Results are presented as averages of 15 min bins during 2 h of environmental cooling **(A,C,E)** and area under the curve (AUC) **(B,D,F)** calculated as the net change from the basal levels before cold exposure. Light phase began at Zeitgeber Time 0 (ZT0) and dark phase (shaded areas) began on ZT12. Results are presented as mean ± SEM * indicates differences between the first and the other time-points within the control or the cold-exposed group, *p* < 0.05. ^#^ indicates differences between control and cold conditions, *p* < 0.05*. n* = 7–11/group/ZT.

The respiratory quotient, an index of substrate oxidation prevalence, decreased during every cold exposure, an effect that was modulated by time-of-day (Figures 5A,B; Table 3). In line with a decrease in RER, cold exposure evoked a consistent increase in lipid oxidation (Figures 5E,F). Interestingly, the higher lipid oxidation’s AUC was apparent only at ZT7–9, 11–13, 15–17, and 19–21, indicating that this rise was accentuated during the dark phase (Figure 5F). Although carbohydrate (CHO) oxidation was not significantly affected by cold exposure, it showed a time-of-day association (Figures 5C,D; Table 3). Increased CHO oxidation was observed only during the ZT3–6 protocol and the first hour of the ZT11–14 protocol (*p* < 0.001). On the other hand, CHO oxidation decreased when animals were exposed to cold during the dark phase, especially at ZT15–18 and 19–22 (*p* < 0.05), when also the largest decreases in RER were observed.

### Effect of Time-of-Day on Clock Genes mRNA Expression in Skeletal Muscle and BAT After Acute Environmental Cooling

The mRNA expression of eight clock genes was investigated in both soleus muscle and BAT (Tables 4 and 5; Figure 5). Time-of-day affected the expression of five of eight and six of eight clock genes in soleus muscle and BAT, respectively. Expression of CRY2 and CLOCK genes was not significantly affected by time-of-day in either tissue, although CLOCK gene expression showed a tendency in the BAT (*p* = 0.054). Cold exposure caused a significant change in most (seven of eight) of the clock genes studied in BAT and half of the clock genes studied in the soleus muscle. In the BAT, time-of-day interacted with cold exposure only for DBP and REV-ERBα expression. In soleus muscle, time-of-day interacted with cold exposure for clock gene expression of five of eight of the genes studied (CLOCK, PER2, CRY1, CRY2, and REV-ERBα). BMAL-1 and DBP were significantly affected by cold exposure in the BAT (*p* < 0.001 and *p* < 0.0001), but not in soleus muscle (*p* = 0.479 and *p* = 0.313). While BMAL-1 was increased by cold exposure especially during the light phase, DBP decreased after the ZT7–10 and ZT11–14 protocols. The negative repressor loop of the core clock (PER1, PER2, CRY1, and CRY2) was upregulated in both BAT and soleus muscle. Interestingly, time-of-day interacted with the increased mRNA expression in three of four of the genes in soleus muscle, but with none of the genes in BAT. Finally, REV-ERBα mRNA expression showed a strong interaction effect of time-of-day and cold exposure in both tissues. In fact, except for ZT3–6, REV-ERBα expression was significantly decreased by cold exposure, with a steep reduction after ZT7–10 in both tissues.

**Table 4:** Significance levels observed for the two-way ANOVA analysis of gene expression in the brown adipose tissue.

	*n*	Time-of-day	Cold exposure	Interaction
BMAL-1	80	<0.0001****	0.0002***	0.4584
CLOCK	79	0.0542	0.0018**	0.4676
PER1	82	0.0657	<0.0001****	0.4003
PER2	80	<0.0001****	<0.0001****	0.1492
CRY1	82	0.0003***	<0.0001****	0.0580
CRY2	83	0.2550	<0.0001****	0.3824
DBP	82	<0.0001***	<0.0001****	<0.0001****
REV-ERBα	8C	<0.0001***	0.3983	0.0018**
CREB	83	0.3688	0.0010**	0.2376
PGCl-α	83	0.0022**	<0.0001****	0.0024**
PGC1-β	81	0.0673	0.8981	0.0568
PPAR-α	82	0.0196*	0.3069	0.0592
PPAR-γ	83	0.1241	0.1606	0.1145
HSP90	83	0.5366	<0.0001****	0.0321*
UCP1	82	0.0043**	<0.0001****	0.3767
CIRP	81	<0.0001****	<0.0001****	<0.0001****
CPT1-β	81	0.2195	0.0011**	0.2755
AMPK	82	0.0402*	0.0053**	0.2437
FAT/CD36	81	0.2195	0.0038**	0.3579
GLUT4	83	0.4685	0.0310*	0.4725
HSL	81	0.0203*	0.0390*	0.2161
LPL	80	0.0756	0.0001****	0.4909
ADR-β3	79	0.0572	0.0412*	0.2272
GR	82	0.2009	0.0506	0.2960
ACC1	73	0.4955	0.0272*	0.5446
ACC2	79	0.4957	0.3617	0.0221*

*p < 0.05; **p < 0.01; ***p < 0.001; ****p < 0.0001.

**Table 5:** Significance levels observed for the two-way ANOVA analysis of the gene expression in the *soleus* muscle.

	*n*	Time-of-day	Cold exposure	Interaction
BMAL-1	80	<0.0001****	0.4785	0.6391
CLOCK	83	0.1868	0.0817	0.0488*
PER1	80	0.0010**	<0.0001****	0.2543
PER2	78	<0.0001****	<0.0001****	0.0183*
CRY1	81	<0.0001****	<0.0001****	0.0199*
CRY2	82	0.3535	0.0002***	0.0016**
DBP	81	<0.0001****	0.3126	0.1582
REV-ERBα	76	<0.0001****	0.7068	0.0308*
CREB	83	0.7026	0.0229*	0.0521
PGC1-α	80	<0.0001****	<0.0001****	0.0006***
PGC1-β	79	0.3707	0.0297*	0.0984
PPAR-α	82	0.0340*	0.0169*	0.0136*
PPAR-γ	80	0.7800	0.0023**	0.3825
HSP90	78	0.0401*	0.1641	0.0015**
UCP2	65	0.5029	0.0189*	0.5634
UCP3	77	<0.0001****	0.9447	0.0009***
CIRP	83	0.7462	0.4239	0.2013
CPT1-α	79	0.0084**	0.0009***	0.0285*
CPT1-β	82	0.1321	0.3225	0.0004***
AMPK	83	0.4686	0.0008***	0.0219*
NAMPT-1	82	0.0655	0.0071**	0.0234*
FAS/CD36	81	0.5285	0.0830	0.0067**
GLUT4	81	0.0071**	<0.0001****	0.0062**
HSL	80	0.3865	<0.0001****	0.1707
LPL	83	0.4184	0.0200*	0.0125*
ADR-β2	82	0.0170*	<0.0001****	0.2198
GR	83	0.3263	<0.0001****	0.0642
FOXO-1	80	0.3576	0.0005***	0.3377
ACC2	13	0.3244	0.0300*	0.0919
mTOR	74	0.5338	0.3857	0.2628
CamK2a	79	0.7143	0.0104*	0.3953

*p < 0.05; **p < 0.01; ***p < 0.001; ****p < 0.0001.

### Effect of Time-of-Day on Metabolic Genes mRNA Expression in BAT After Acute Environmental Cooling

**Figure 6:**
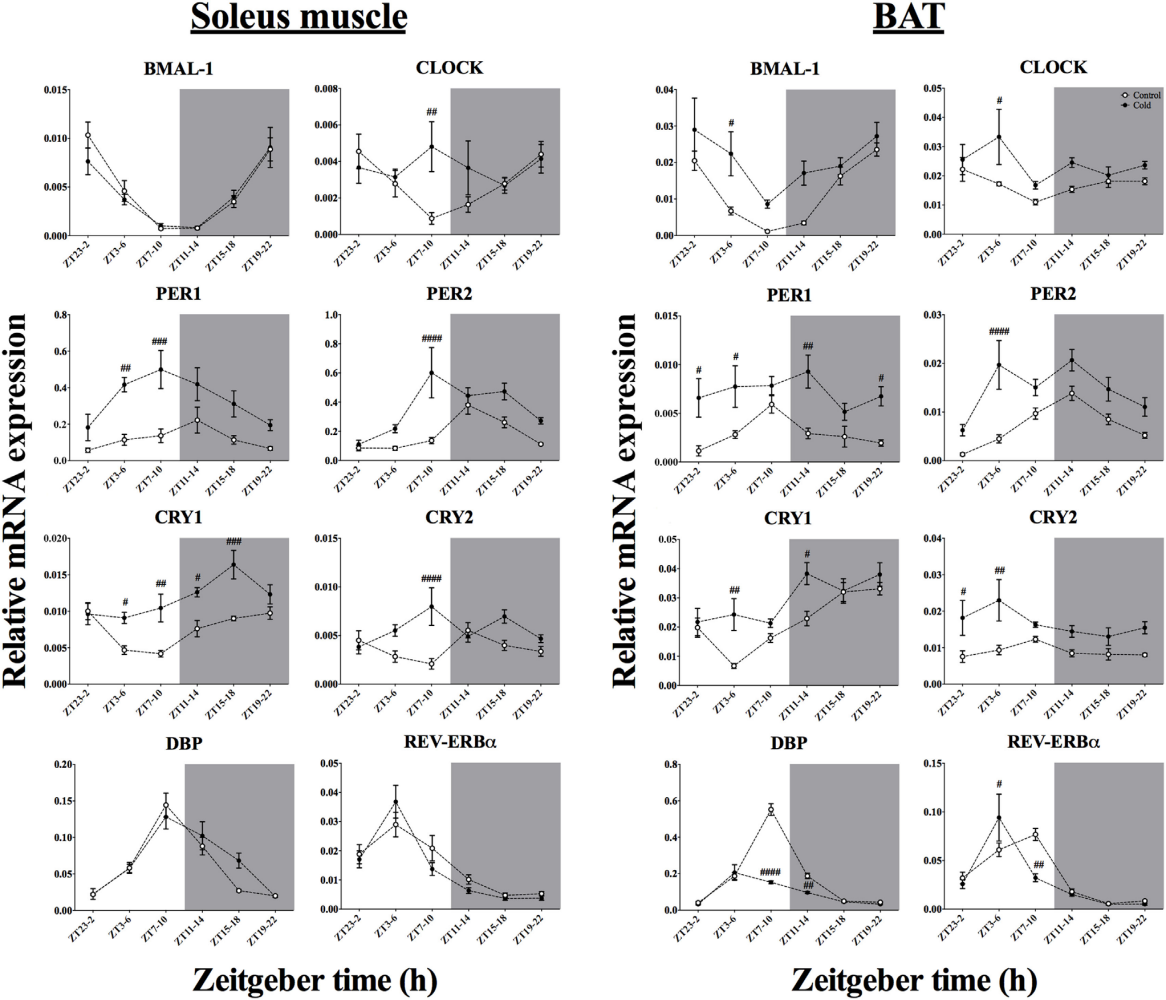
Effect of time-of-day on clock gene expression in both soleus muscle and brown adipose tissue (BAT) after exposure to environmental cooling. Light phase began at Zeitgeber Time 0 (ZT0) and dark phase (shaded areas) began at ZT12. Results are presented as mean ± SEM. *p* < 0.05. ^#^ indicates differences between control and cold conditions, *p* < 0.05*. n* = 5–7/group/ZT.

We studied 18 genes directly or indirectly related to metabolism or thermogenesis in the BAT (Table 4; Figure 6). Time-of-day affected the mRNA expression of 6 of 18 of the genes studied (PGC1-α, PPAR-α, UCP1, CIRBP, AMPK, and HSL) and just missed significance in 3/18 (PGC1-β, LPL, and ADR-β3). Cold exposure affected most of the BAT genes studied (13 of 18 genes) and just missed significance for GR mRNA expression (*p* = 0.051). Only 4 of 18 of the investigated genes showed a significant interaction between time-of-day and cold exposure (PGC1-α, HSP90, CIRBP, and ACC2), while PGC1-β and PPAR-α almost reached significance. The genes that were most clearly affected by cold exposure were PGC1-α, HSP90, UCP1, CIRBP, and LPL *(p* < 0.0001).

### Effect of Time-of-Day on Metabolic Genes mRNA Expression in Skeletal Muscle After Acute Environmental Cooling

**Figure 7:**
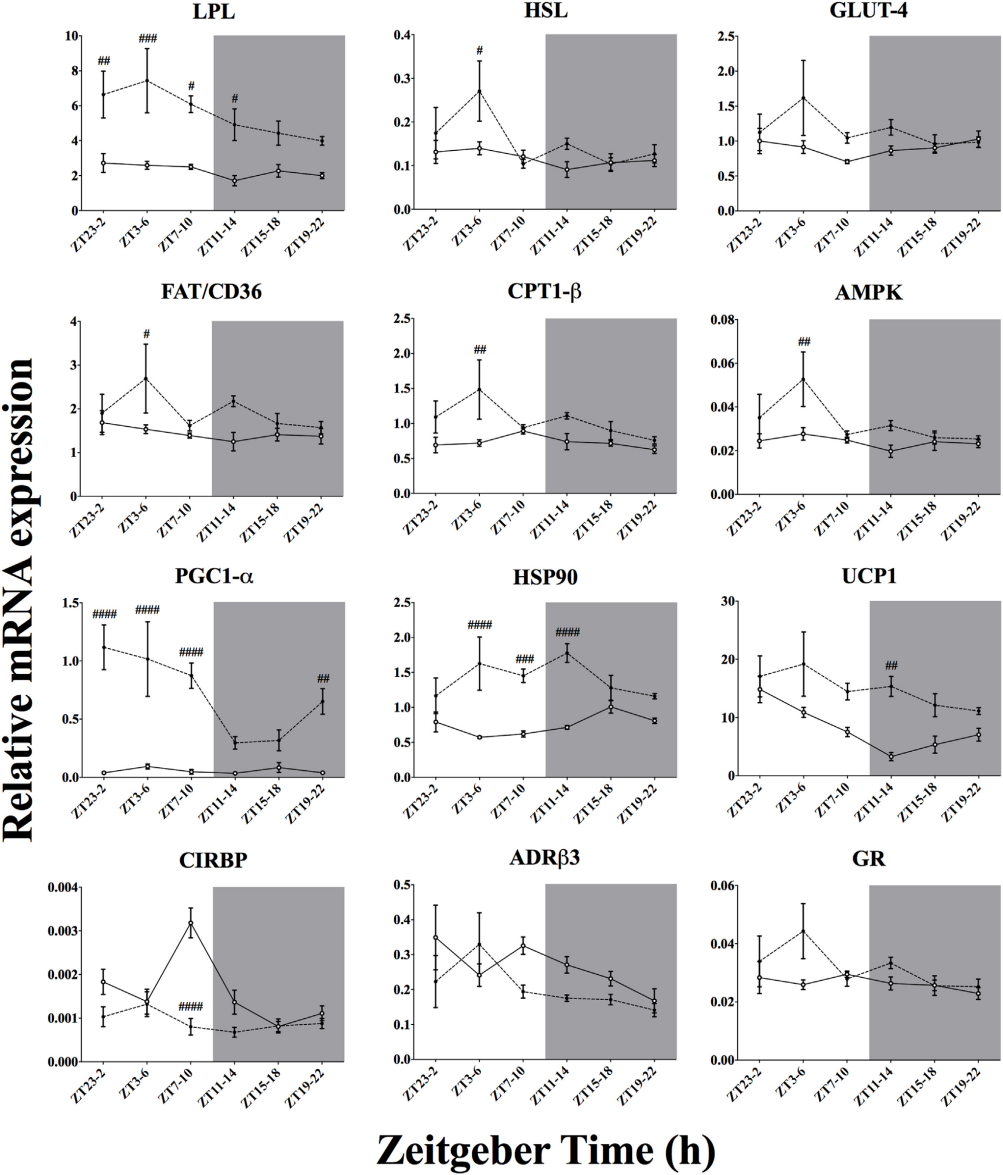
Effect of time-of-day on metabolic gene expression in brown adipose tissue (BAT) after exposure to environmental cooling. Light phase began at Zeitgeber Time 0 (ZT0) and dark phase (shaded areas) began at ZT12. Results are presented as mean ± SEM. ^#^ indicates differences between control and cold conditions, *p* < 0.05*. n* = 5–7/group/ZT. Data for CREB, PGC1-β, PPAR-α, PPAR-γ, ACC1, and ACC2 are not shown (refer to Table 4 for results).

We studied 23 genes directly or indirectly related to metabolism or thermogenesis in the soleus muscle (Table 5; Figure 7). Time-of-day affected the mRNA expression of 7 of 23 genes (PGC1-α, PPAR-α, HSP90, UCP3, CPT1-α, GLUT4, and ADR-β2) and just missed significance for NAMPT-1 expression (*p* = 0.066). Cold exposure affected mRNA expression of 17 of 23 of the studied genes and almost reached significance for FAT/CD36 levels (*p* = 0.083). The interaction between time-of-day and cold exposure was significant for 11 of 23 of the studied genes and just missed significance for 4 of 23 genes (CREB, PGC1-β, GR, and ACC2). The genes that were most clearly affected by cold exposure were PGC1-α, GLUT4, HSL, ADRβ2, and GR *(p* < 0.0001).

## Discussion

The main finding of the present study is that the cold-induced metabolic response and changes in gene expression in BAT and muscle differ depending on the time-of-day of the cold exposure. For instance, the cold-induced increase in lipid oxidation was mainly observed during the dark phase (Figures 5E,F), indicating a daily modulation of the cold-induced metabolic adaptations. In the soleus skeletal muscle, particularly active during shivering thermogenesis, cold exposure increased the expression of clock genes in both the negative and positive loop of the core clock mechanism, PER/CRY and BMAL-1/CLOCK, respectively (Figure 6). In addition, besides upregulating clock gene expression in both regulatory loops of the BAT molecular clock as well, cold exposure inhibited the expression of DBP and REV-ERBα in BAT (Figure 6). The current findings add further evidence for a tissue-specific action of the internal clock in peripheral tissues such as the skeletal muscle and BAT. Whether this differential modulation in response to environmental *stimuli* relies on the activity of the central clock, extra-SCN sites within the central nervous system or peripheral inputs remains to be further elucidated.

### Effects of Cold Exposure During Different Times-of-Day on Thermogenesis and Substrate Oxidation

The thermal and metabolic adjustments induced by an acute exposure to a cold environment are well known for mice (24–29), rats (6, 8, 30–32), and humans (9, 33–35). Our experiments confirmed the effects of cold on the induction of increased heat production mostly through the concerted modulation of behavioral (LA, food, and water intake) and metabolic (T_core_, VO_2_, RER, EE, CHO, and lipid oxidation) processes. In accordance with our hypothesis, we found that time-of-day modulated the thermal, behavioral, and metabolic responses.

Significant decrements of ambient temperature pose a primitive threat to body temperature regulation. In order to keep body temperature within physiological levels, the central nervous system modulates a number of physiological processes involved in heat conservation and production. In the present experiment, we observed that rat T_core_ is resilient to a temporally limited exposure to a reduced ambient temperature. In fact, after 120 min of environmental cooling, normal T_core_ was preserved for each time-of-day exposure (Figure 3A). Curiously, we even detected a small rise in T_core_ when the animals were challenged with a cold environment presented during the light phase, an effect that is not observed in smaller rodents, such as mice (24–29). This mismatch of heat production and heat dissipation (leading to body heating) might be due to a heat defensive state potentiated by the sudden decrease in ambient temperature during the sleep period.

Indeed, a lower T_core_ during the light phase is maintained through tail vasoconstriction and decreased EE (36–39). In such a condition, the T_core_ regulatory system likely presents a higher sensitivity to changes in locomotor and metabolic activity (40), resulting in increased T_core_ during the first hour of cold exposure in the light phase, when heat production was increased (Figure 3A). Conversely, during the dark phase, basal T_core_ is slightly raised as a function of the increased LA, EE, and circadian rhythm (39), making the immediate impact of the increased metabolic rate on T_core_ less perceptible.

Indeed, the well-described circadian-dependent decrease in T_core_ during the second half of dark phase seems to be counterbalanced by heat production induced by cold exposure (Figure 3A, ZT15–17). This cold-induced hyperthermia was also reported by others (6, 31, 41–43). Initially, heat dissipation is reduced to minimize heat loss to the colder environment (6). As cold exposure is maintained, heat production is increased and heat loss and production reach a steady state in which T_core_ can be successfully preserved. Therefore, as previously observed with other stressors (40, 44–47), it seems that the time-of-day-dependent effects of cold exposure on body temperature reflect the transitory disturbance in heat loss and heat production mechanisms elicited by mixed signals from the internal circadian time and the thermoregulatory pathways that participate in the homeostatic responses.

In line with this perspective, the increased metabolic rate (Figures 3C,D) and LA (Figures 4A,B) in response to cold exposure might also reflect environmental temperature gradient (intensity of the stimulus) rather than a time-of-day-dependent modulation. Interestingly, LA spiked during the first hour of the cooling protocol but decreased thereafter (Figure 4A). Similar results were described by others (43, 48, 49), suggesting that this probably reflects coupled mechanisms of heat conservation (cold avoidance behavior reflecting increased LA) and heat production (shivering thermogenesis reflecting the decreased LA). In fact, as the environment gets colder, the animals lessen their LA without dampening metabolic rate and EE (Figures 3C,E), which points toward increased shivering activity of thermogenic pathways. Moreover, both oxygen consumption and calculated EE reached steady states of increased activity regardless of time-of-day. This is in accordance with previous experiments in mice showing that the relative changes in metabolic rate were similar when the cooling protocol started at the beginning of the light or dark phase (27, 29). Interestingly, Tokizawa and colleagues (29) observed that the threshold for increased heat production was elevated during the dark phase, while behavioral curling was elicited earlier during the light phase. In rats, a similar effect of time-of-day on thermoregulatory thresholds was also observed during exercise (47). To our knowledge, this is the first time that a time-of-day effect on the cold defensive response is reported for the rat model. With the present results, it seems that the circadian system regulates the basal settings of body temperature, thereby indirectly establishing the level of the homeostatic response required to minimize or even neutralize the physical challenge on thermal homeostasis posed by the reduced ambient temperature.

Even though thermal balance was successfully defended during cold exposure regardless of time-of-day, lipid and CHO utilization were affected by both environmental time and temperature (Figure 5). It has been previously shown that environmental cooling provides a key signal to substrate utilization for shivering (involuntary muscle contractions) and non-shivering (generated by proton gradient within mitochondrial oxidative activity and controlled by uncoupling proteins) thermogenesis from skeletal muscle and BAT, respectively (6, 8, 30, 50–52). Environmental conditions and the intensity of cold exposure determine the metabolic rate and the prevalence of the thermogenic pathway activated to generate heat (6, 8, 51). Vaillancourt and colleagues (8) have previously shown that although affecting total EE; below a certain threshold (~15°C), the higher rates of CHO, lipid, and protein oxidations are not intensified by even lower environmental temperatures (10 and 5°C). To our knowledge, we are the first to report a clear time-of-day effect on substrate utilization during cold exposure.

Interestingly, we observed that during the active period, cold exposure caused a major switch by increasing lipid oxidation (Figures 5E,F) and lessening CHO oxidation (Figures 5C,D), whereas during the light phase both lipid and CHO oxidation rates were increased, although only transitory for CHO. This might reflect the first stage of cold defensive mechanisms in which in skeletal muscle shivering first consumes carbohydrates stocks in the glycolytic pathway to promptly generate ATP for muscular contraction and heat production (51, 52). As cold exposure persists, the participation of shivering in the thermal balance decreases and a primary role is appropriated by BAT non-shivering thermogenesis, with increased lipid uptake and oxidation (35, 52). Therefore, time-of-day influences the balance between CHO and lipids utilization during cold exposure, providing further evidence that the daily rhythms expressed through an internal clock in different body tissues might influence metabolic adjustments in heat-generating tissues.

### Differences in the Metabolic and Thermal Adjustments to Cold Were Related to Distinct Changes in mRNA Expression of Clock and Clock-Controlled Genes

**Figure 8:**
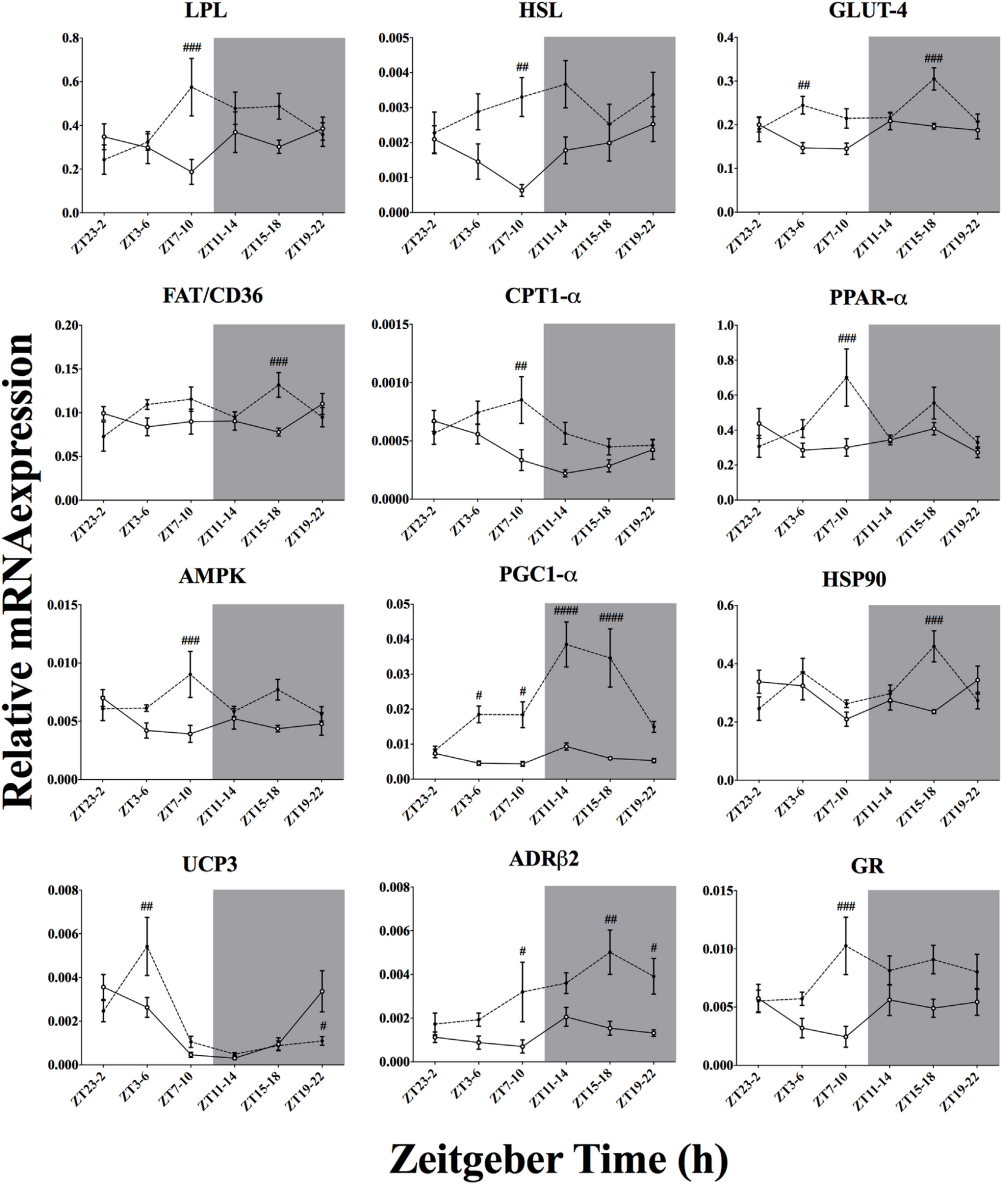
Effect of time-of-day on metabolic gene expression in soleus muscle after exposure to environmental cooling. Light phase began at Zeitgeber Time 0 (ZT0) and dark phase (shaded areas) began at ZT12. Results are presented as mean ± SEM. ^#^ indicates differences between control and cold conditions, *p* < 0.05*. n* = 5–7/group/ZT. Data for CREB, PGC1-β, PPAR-γ, *UCP2*, CIRBP, *CPT-β*, NAMPT-1, FOXO-1, ACC2, CamK2a, and *mTOR* are not shown (refer to Table 5 for results).

To increase metabolic heat production during exposure to a lower environmental temperature, activity in skeletal muscle and BAT is intensified through different pathways within the central nervous system (6). In the present study, we observed that after 3 h of cold exposure 13 of 18 and 17 of 23 of the chosen genes thought to be involved in cold-induced metabolic heat production were affected by our cold protocol in the BAT (Figure 7) and skeletal muscle (Figure 8), respectively.

In addition to the metabolic effects, cold exposure also affected clock and clock-controlled gene expression in BAT and soleus muscle, although to a different extent (Figure 6). In the BAT, seven of eight core clock genes studied were altered by the lower environmental temperature. Specifically, five of those genes were upregulated after cold exposure, without an interaction with time-of-day on this response. Others have already implicated PER1, PER2, CRY1, and CRY2 in BAT in cold exposure (12, 13). In fact, a functional clock is necessary to mediate molecular adjustments in the mitochondrial activity program (13, 17, 53, 54). However, the present study is the first to show this upregulation of BAT activity during cold exposure at different moments of the day, reinforcing the hypothesis that the thermogenic program, although it is intermingled with the core clock machinery (55), relies on the intensity of the stimulus rather than on time-of-day.

Interestingly, in BAT two of the clock genes, DBP and REV-ERBα, were downregulated by cold exposure with a time-of-day interaction. REV-ERBα is also downregulated after increased energetic demands and is acknowledged as an important repressor of mitochondrial activity and seems to be regulated by the positive loop of the core clock (16, 17, 53). In fact, we observed that the reduced REV-ERBα expression is accompanied by an augmented expression of BMAL-1 and PGC1-α, especially during the light phase (Figures 6 and 7), which points toward a pronounced shift in BAT mitochondrial activity in response to the light-to-dark transition (54) and change in ambient temperature, probably through a temperature compensation mechanism.

Regarding the soleus skeletal muscle, we observed that only the genes from the negative loop of the canonical core clock machinery were affected by cold exposure (Figure 6; Table 5), i.e., PER1, PER2, CRY1, and CRY2, that is half of the studied clock genes (four of eight). In line with this, resetting of the peripheral clock by cold exposure (13) and exercise (56) seems to be dependent of *Per1/2* (57), as is resetting by glucocorticoids fluctuations (58). These findings reinforce the idea that cold exposure can reset the peripheral clock by changes in metabolic activity, since genes encoding for mitochondrial activity, such as CPT1, PGC1α, PPAR, and FOXO1 were affected. In addition, genes that regulate substrate uptake (LPL and GLUT4), cellular energetic state (AMPK), and mitochondrial activity (CPT1, NAMPT, PGC1α and PPARs) showed an interaction with time-of-day, suggesting that key components of the cellular energetic balance are dependent on temporal input to establish the necessary shifts in molecular programs required to survive in a colder environment. On the other hand, cold exposure also changed GR and ADRβ receptor expression in both soleus muscle and BAT, indicating that also hormonal and autonomic, i.e., non-metabolic, factors might be involved in this shifting process.

In the skeletal muscle (Figure 8), the increased expression of LPL, HSL, and FAT/CD36 points toward boosted lipid uptake, which is supported by previous physiological and molecular expression data (59). Glucose uptake was also elevated by cold exposure as suggested by increased GLUT4 expression in the present experiment and those performed by others (10, 11). Taken together, increased lipid and glucose uptake suggests an augmented substrate oxidative state within the soleus muscle. Reinforcing this assumption, the expression of key metabolic regulators such as AMPK, CamK2a, and NAMPT-1 was also increased after cold exposure (Table 5). It is thought that AMPK activation due to increased energetic demand regulates the key mitochondrial transporter for β-oxidation, CPT1-α/β, as well as key transcription factors for mitochondrial activity, such as PPARs, PGC1-α, and FOXO1 (60–65). In fact, all these genes associated with mitochondrial activity were upregulated in the skeletal muscle. The present gene expression results thus support a role for the skeletal muscle in metabolic heat production. This idea is also supported by increased HSP90 mRNA expression, which might be related to higher oxidative activity demanding more chaperone protein content for protein stabilization (66).

Interestingly, despite the higher energetic demand, we did not observe a clear effect of cold exposure on UCP3 activity (Figure 8). It is thought that UCP3 could be directly involved in muscular heat production independent of shivering (35, 67). In line with our results, others (68) have shown that muscular UCP3 activity might not be involved in cold-induced metabolic heat production. Instead, uncoupling seems to constitute an intramuscular FFA buffering system during cold exposure, at least in murine models (12, 69). In addition, we also observed a possible influence of neuronal and hormonal stimulation on these molecular adjustments since expression of ADRβ2 and GR was upregulated after cold exposure, pointing to a direct modulation of the cold-induced adjustments by the sympatho-cortico-adrenal system (12, 70–72).

In the BAT, cold exposure also upregulated the substrate uptake and mobilization program, as can be concluded from the increased mRNA expression of LPL, FAT/CD36, and GLUT4 (Figure 7; Table 4). Transcription of key enzymes for β-oxidation, such as CPT1-β and ACC1 was also upregulated by cold exposure. Aligned with this finding, mitochondrial activity, and biogenesis, expressed by the function of the key transcription factor PGC1α was increased by cold exposure. This increased mitochondrial activity state is reinforced by improved UCP1, increased HSP90 (66) and decreased CIRPB (73) expression after cold exposure. AMPK activity, which is thought to play a key role in mediating cellular metabolic flux, was further stimulated by cold exposure. Increased BAT activity is well supported by previous studies and accumulating evidence implicates both glucose and lipid oxidation in BAT non-shivering thermogenesis (14, 74–77).

It is clear that the initial metabolic state, as defined by the circadian system, is crucial for the physiological response to environmental stimuli and therefore should be taken into account to understand the intrinsic capacity to adapt to changes in ambient temperature. In the current experiment, the cold-induced metabolic shift toward lipid oxidation provided a clear example of such a time-dependency and further proof that the internal clock plays an important role in shaping such physiological responses. In the soleus skeletal muscle, cold exposure upregulated specifically the negative loop of the canonical clock (PER/CRY), whereas in the BAT both the negative and positive loop (BMAL-1/CLOCK) were upregulated. Thereby, the present study for the first time showed a tissue-specific effect of cold exposure on clock and clock-controlled genes and thus demonstrated the existence of an interaction between time-of-day and homeostatic adjustments elicited by acute cold exposure.

## Ethics Statement

This study was carried out in accordance with the recommendations of the animal care committee of the Royal Netherlands Academy of Arts and Sciences (DEC/KNAW). The protocol was approved by the animal care committee of the Royal Netherlands Academy of Arts and Sciences (DEC/KNAW).

## Author Contributions

FM, CC, and AK designed the experiments. FM, ZZ, YS, PG, RJ, and EF acquired the data. FM, ZZ, EF, CC, and AK contributed to the analysis and interpretation of data. FM and AK participated in the elaboration of the manuscript and gave final approval for submission and publication, being accountable for all aspects of the present work.

## Conflict of Interest Statement

The authors declare that the research was conducted in the absence of any commercial or financial relationships that could be construed as a potential conflict of interest.
