# Supplementary material for: Time-of-Day Effects on Metabolic and Clock-Related Adjustments to Cold
Source: Front Endocrinol (Lausanne). 2018 Apr 26;9:199. doi: 10.3389/fendo.2018.00199 (PMC5932155; doi:10.3389/fendo.2018.00199)
Supplement: Supplementary file 1 [file table_1.PDF]

Supplementary Table 1. Similarities of thermal, behavioral and metabolic parameters 24 hours before and immediately before the onse of environmental cooling at six moments of the day (Averages refer to 15 min intervals).

		**ZT23**	**ZT3**	**ZT7**	**ZT11**	**ZT15**	**ZT19**
		Mean ± SD (n)	Mean ± SD (n)	Mean ± SD (n)	Mean ± SD (n)	Mean ± SD (n)	Mean ± SD (n)
**Body temperature (°C)**	Control	37.31 ± 0.26 (5)	36.88 ± 0.36 (8)	37.21 ± 0.11 (6)	37.38 ± 0.13 (3)	37.83 ± 0.34 (5)	37.65 ± 0.29 (8)
	Cold	37.56 ± 0.24 (5)	36.71 ± 0.25 (8)	37.25 ± 0.10 (6)	37.38 ± 0.20 (5)	37.82 ± 0.49 (5)	37.70 ± 0.32 (7)
**LA (Cnts)**	Control	437.43 ± 253.80 (7)	256.43 ± 323.34 (7)	150.91 ± 143.92 (11)	105.57 ± 237.74 (7)	789.29 ± 747.17 (7)	457.18 ± 709.21 (11)
	Cold	697.43 ± 476.32 (7)	348.00 ± 442.88 (7)	360.73 ± 504.89 (11)	340.43 ± 386.24 (7)	837.14 ± 776.36 (7)	571.50 ± 620.44 (4)
**VO2 (ml/h/kg)**	Control	1564.00 ± 272.46 (7)	1319.14 ± 298.92 (7)	1300.64 ± 165.94 (11)	1468.29 ± 254.34 (7)	1813.14 ± 217.04 (7)	1663.36 ± 617.98 (11)
	Cold	1708.43 ± 279.94 (7)	1290.86 ± 204.53 (7)	1420.00 ± 285.98 (11)	1453.00 ± 206.59 (7)	1939.43 ± 259.23 (7)	1796.91 ± 434.24 (11)
**RER (VO2/CO2)**	Control	1.05 ± 0.01 (7)	0.99 ± 0.03 (7)	1.00 ± 0.04 (11)	1.03 ± 0.03 (7)	1.05 ± 0.02 (7)	1.02 ± 0.08 (11)
	Cold	1.04 ± 0.02 (7)	1.00 ± 0.03 (7)	0.97 ± 0.03 (11)	1.00 ± 0.05 (7)	1.04 ± 0.02 (7)	1.05 ± 0.04 (11)
**Energy expenditure (kcal/h/kg)**	Control	7.65 ± 0.67 (7)	6.65 ± 1.54 (7)	6.52 ± 0.92 (11)	7.45 ± 1.25 (7)	9.26 ± 1.09 (7)	8.62 ± 2.89 (11)
	Cold	8.38 ± 1.32 (7)	6.52 ± 1.07 (7)	7.20 ± 1.44 (11)	7.33 ± 1.00 (7)	9.87 ± 1.32 (7)	9.19 ± 2.07 (11)
**Food intake (g)**	Control	0.00 ± 0.00 (7)	0.00 ± 0.00 (7)	0.00 ± 0.00 (11)	0.16 ± 0.42 (7)	0.11 ± 0.28 (7)	0.15 ± 0.43 (11)
	Cold	0.50 ± 1.19 (7)	0.27 ± 0.71 (7)	0.16 ± 0.41 (11)	0.40 ± 0.82 (7)	0.00 ± 0.00 (7)	0.38 ± 0.78 (11)
**Water intake (ml)**	Control	0.20 ± 0.37 (7)	0.00 ± 0.00 (7)	0.01 ± 0.02 (11)	0.00 ± 0.01 (7)	0.69 ± 0.79 (7)	0.24 ± 0.72 (11)
	Cold	0.27 ± 0.53 (7)	0.01 ± 0.02 (7)	0.12 ± 0.33 (11)	0.00 ± 0.01 (7)	0.45 ± 0.56 (7)	0.08 ± 0.14 (11)
