# Supplementary material for: Time-of-Day Effects on Metabolic and Clock-Related Adjustments to Cold
Source: Front Endocrinol (Lausanne). 2018 Apr 26;9:199. doi: 10.3389/fendo.2018.00199 (PMC5932155; doi:10.3389/fendo.2018.00199)
Supplement: Supplementary file 2 [file table_2.docx]

| **Supplementary Table 2.** Averages and ANOVA one-way table demonstrating the effect of ZT on net area under the curve (AUC, in arbitrary units for each parameter) for thermal, behavioral and metabolic parameters under control or cold exposure (Mean ± SD (n)). | | | | | | | | | | | | | | | | | | | | | |
| --- | --- | --- | --- | --- | --- | --- | --- | --- | --- | --- | --- | --- | --- | --- | --- | --- | --- | --- | --- | --- | --- |
|  |  |  |  |  |  |  |  |  |  |  |  |  |  |  |  |  |  |  |  |  |  |
|  |  | **ZT23** | | | **ZT3** | | | **ZT7** | | | **ZT11** | | | **ZT15** | | | **ZT19** | | | ZT effect | *p value* |
|  |  | Mean ± SD (n) | | | Mean ± SD (n) | | | Mean ± SD (n) | | | Mean ± SD (n) | | | Mean ± SD (n) | | | Mean ± SD (n) | | |  |  |
| Tcore | control | 3.62 | ± | 33.25 (5) | 2,16 | ± | 22.78 (5) | -3,97 | ± | 14.99 (6) | 0,28 | ± | 14.97 (5) | -12,67 | ± | 42.94 (5) | 17,92 | ± | 20.41 (8) | F(5,28)=2.78 | *0,439* |
|  | cold | -19,62 | ± | 35.08 (5) | 43,75 | ± | 12.42 (5) | 30,23 | ± | 34.44 (6) | 39,77 | ± | 34.41 (5) | 35,28 | ± | 37.78 (5) | 18,68 | ± | 29.10 (8) | F(5,28)=0.99 | 0.037* |
| LA | control | 1525 | ± | 33917 (7) | -17942 | ± | 20131 (7) | 8350 | ± | 10089 (11) | 26624 | ± | 21810 (7) | -10451 | ± | 51077 (7) | 15974 | ± | 41892 (4) | F(5,37)=2.00 | 0,101 |
|  | cold | 71690 | ± | 59710 (7) | 73450 | ± | 32851 (7) | 60249 | ± | 42136 (11) | 71811 | ± | 55027 (7) | 77176 | ± | 78436 (7) | 54348 | ± | 49389 (4) | F(5,37)=0.16 | 0,974 |
| VO2 | control | -6550 | ± | 34505 (7) | -6370 | ± | 29205 (7) | 7546 | ± | 17551 (11) | 19952 | ± | 19213 (7) | 469 | ± | 45909 (7) | 5546 | ± | 52282 (11) | F(5,44)=0.55 | 0,741 |
|  | cold | 83619 | ± | 41958 (7) | 109659 | ± | 26355 (7) | 110883 | ± | 25571 (11) | 123505 | ± | 18243 (7) | 84484 | ± | 36144 (7) | 92478 | ± | 56659 (11) | F(5,44)=1.39 | 0,247 |
| RER | control | -3,02 | ± | 0.70 (7) | -2,32 | ± | 1.25 (7) | -0,89 | ± | 0.68 (11) | -0,08 | ± | 0.54 (7) | -0,49 | ± | 1.18 (7) | 0,58 | ± | 1.55 (11) | F(5,44)=1.41 | 0,239 |
|  | cold | -10,72 | ± | 1.52 (7) | -8,17 | ± | 1.42 (7) | -4,864 | ± | 1.48 (11) | -10,232 | ± | 2.55 (7) | -16,31 | ± | 1.92 (7) | -15,67 | ± | 2.37 (11) | F(5,44)=5.47 | 0.0005*** |
| EE | control | -32 | ± | 169 (7) | -35 | ± | 146 (7) | 36 | ± | 87 (11) | 100 | ± | 96 (7) | 0,46 | ± | 227 (7) | 31 | ± | 250 (11) | F(5,44)=0.59 | 0,704 |
|  | cold | 392 | ± | 212 (7) | 540 | ± | 79 (7) | 539 | ± | 131 (11) | 588 | ± | 95 (7) | 377 | ± | 174 (7) | 419 | ± | 271 (11) | F(5,44)=1.89 | 0,116 |
| FI | control | 15,41 | ± | 18.28 (7) | 9,25 | ± | 11.32 (7) | 13,94 | ± | 8.56 (11) | 14,3 | ± | 31.71 (7) | 15,72 | ± | 57.70 (7) | 33,64 | ± | 28.55 (11) | F(5,43)=0.71 | 0,569 |
|  | cold | 30,53 | ± | 18.05 (6) | 37,17 | ± | 11.16 (7) | 40,16 | ± | 26.26 (11) | 36,8 | ± | 61.44 (7) | 22,67 | ± | 61.08 (7) | 12,65 | ± | 68.92 (11) | F(5,43)=0.46 | 0,801 |
| WI | control | 12,5 | ± | 32.70 (7) | 5,91 | ± | 17.89 (7) | 1,43 | ± | 4.45 (11) | 30 | ± | 18.99 (7) | -12,65 | ± | 64.15 (7) | 36,25 | ± | 50.44 (11) | F(5,44)=2.08 | 0,085 |
|  | cold | 10,96 | ± | 56.81 (7) | 14,8 | ± | 9.71 (7) | 10,22 | ± | 20.07 (11) | 33,74 | ± | 21.53 (7) | 4,27 | ± | 42.30 (7) | -10,84 | ± | 81.75 (11) | F(5,44)=0.76 | 0,581 |
| Lipid Oxidation | control | 0,12 | ± | 0.07 (7) | 0,08 | ± | 0.10 (7) | 0,06 | ± | 0.12 (11) | 0,01 | ± | 0.05 (7) | 0,05 | ± | 0.15 (7) | -0,06 | ± | 0.98 (11) | F(5,44)=0.16 | 0,975 |
|  | cold | 0,75 | ± | 0.32 (7) | 0,59 | ± | 0.20 (7) | 0,75 | ± | 0.60 (11) | 0,87 | ± | 0.61 (7) | 1,26 | ± | 0.43 (7) | 1,3 | ± | 0.68 (11) | F(5,44)=2.64 | 0.036* |
| CHO Oxidation | control | -0,58 | ± | 0.74 (7) | -0,32 | ± | 0.67 (7) | 0,02 | ± | 0.36 (11) | 0,5 | ± | 0.48 (7) | -0,07 | ± | 0.68 (7) | 0,31 | ± | 1.75 (11) | F(5,44)=1.26 | 0,299 |
|  | cold | -0,29 | ± | 0.95 (7) | 0,73 | ± | 0.57 (7) | 0,38 | ± | 1.48 (11) | 0,33 | ± | 1.60 (7) | -1,54 | ± | 0.96 (7) | -1,47 | ± | 1.21 (11) | F(5,44)5.58 | 0.0005*** |
